# Recent advances in managing and understanding nephrolithiasis/nephrocalcinosis

**DOI:** 10.12688/f1000research.7126.1

**Published:** 2016-04-18

**Authors:** Giovanni Gambaro, Alberto Trinchieri

**Affiliations:** 1 Nephrology Division, Department of Internal Medicine and Medical Specialties, Columbus-Gemelli University Hospital, Rome, Italy; 2 Urology Unit, Manzoni Hospital, Lecco, Italy

**Keywords:** nephrolithiasis, nephrocalcinosis, Urinary stone disease, renal stone, nephron

## Abstract

Urinary stone disease is a very common disease whose prevalence is still increasing. Stone formation is frequently associated with other diseases of affluence such as hypertension, osteoporosis, cardiovascular disease, metabolic syndrome, and insulin resistance. The increasing concentration of lithogenic solutes along the different segments of the nephron involves supersaturation conditions leading to the formation, growth, and aggregation of crystals. Crystalline aggregates can grow free in the tubular lumen or coated on the wall of the renal tubule. Plugs of crystalline material have been highlighted in the tubular lumen in some patients, but crystalline growth starting from plaques of calcium phosphate within the renal papillae has been demonstrated in others. Urinary supersaturation is the result of a complex interaction between predisposing genetic features and environmental factors. Dietary intake is certainly the most important environmental risk factor. In particular, an insufficient intake of dietary calcium (<600 mg/day) can increase the intestinal absorption of oxalate and the risk of calcium oxalate stone formation. Other possible risk factors that have been identified include excessive intake of salt and proteins. The potential role of dietary acid load seems to play an important role in causing a state of subclinical chronic acidosis; therefore, the intake of vegetables is encouraged in stone-forming patients. Consumption of sugar-sweetened soda and punch is associated with a higher risk of stone formation, whereas consumption of coffee, tea, beer, wine, and orange juice is associated with a lower risk. A high fluid intake is widely recognized as the cornerstone of prevention of all forms of stones. The effectiveness of protein and salt restriction has been evaluated in some studies that still do not allow definitive conclusions to be made. Calcium stone formation can be prevented by the use of different drugs with different mechanisms of action (thiazide diuretics, allopurinol, and potassium citrate), but there is no ideal drug that is both risk free and well tolerated.

## Introduction

Urinary stone disease is the formation or the presence of concretions in the urinary tract. Stones have different compositions (mainly calcium) and tend to recur, requiring iterative treatments. Urinary stone disease is very common, and its prevalence is still increasing. At least one in ten of the potential readers of this article has already experienced a stone episode or could experience it during their lives. This global epidemic of the disease is even more relevant because it is associated with the increasing prevalence of other non-communicable diseases as the result of the epochal changes in the lifestyle of the world's population.

Urolithiasis is the result of a complex interaction between genetic and environmental factors, and current research is oriented in these two directions, which should enable us to study genetic aspects in combination with environmental exposures. Another unique aspect of this fascinating field of research is the convergence of interests of various specialists in a single subject. Researchers from different backgrounds (chemists, epidemiologists, dieticians, geneticists, pathologists, radiologists, nephrologists, and urologists) come together at the same conferences, they publish in the same magazines and write books together, and they form what we jokingly call "the stone family".

The most important aspects of this research are the epidemiology of the disease, the association with other diseases, the genetics behind the disease, the influence of diet and other environmental factors, and the different ways of preventing the disease.

## Epidemiology

Over the past 20 years, the world epidemiology of urolithiasis has undergone major changes ^1– 4^ related to several factors, such as aging of the population and changes in the lifestyle of the female youth population (low intake of fruits and vegetables and higher consumption of simple sugars and foods with high protein and salt content) ^5^ in Western countries and the westernization of dietary habits and lifestyle in developing countries. In the countries of northern Europe and America, the prevalence values reached a plateau in the late 1980s up to values of 15–20%. Subsequently, no further increases were recorded, although changes were observed in the distribution between males and females and the age of onset of the disease. The prevalence of the disease in women tends to rise and to equal that in men as a result of the more frequent onset of the disease among women aged <30 years. Calcium oxalate is still by far the most frequent composition of urinary calculi, but also uric acid calculi tend to increase as a result of aging of the population and increasing penetration of the metabolic syndrome. On the contrary, infection stones tend to be less frequent as a result of improving health in the population and better methods of treatment of kidney stones. In non-Western countries, particularly in North Africa, the Middle East, India, and China, the prevalence of urolithiasis is increasing with the change of lifestyle due to the improvement of socio-economic conditions and globalization. The warmer climate in the so-called "stone belt" contributes to the rapid increase in prevalence in these regions. Global warming could pose a global climate change that could further increase the prevalence of urolithiasis, even in areas with a more temperate climate.

## Related diseases

Renal stone formation is frequently associated with other diseases of affluence. Calcium stone formation characterized by alterations in the metabolic regulation of calcium and sodium is frequently associated with hypertension, osteoporosis, and cardiovascular disease, whereas uric acid stones (but to some extent calcium stones too) are often linked to the metabolic syndrome and insulin resistance. In a large cohort of >50,000 men, the risk of hypertension was increased in renal stone formers (odds ratio [OR] 1.31) ^6^, and in another cohort of 90,000 women the risk of a new diagnosis of hypertension was higher in subjects with a history of nephrolithiasis (relative risk [RR] 1.36) ^7^. The link between the two diseases has been identified in common alterations of calcium metabolism ^8^. A history of renal stones was associated with lower bone mineral density in a cross-sectional study of 5995 men >65 years old ^9^, and in another national cross-sectional study in the US, bone mineral density of the femoral neck was found to be lower in men with renal stone history after adjusting for age, body mass index, race, and other potential confounders ^10^. In large cohorts of renal stone formers, the risk of myocardial infarction (+31-78%), angina (+61%), and carotid artery atherosclerosis (+60%) was increased, even after adjustment for other known risk factors ^11– 14^. Renal stone formers have increased arterial stiffness, higher arterial calcification score, and reduced bone density ^15, 16^, a complex of findings which has been observed in other conditions—hypertension, osteoporosis, and chronic kidney disease (CKD)—and which may suggest that the vessel wall acts as a buffer for the excessive quantity of calcium coming from high bone turnover. The increased arterial calcification and stiffness may explain the higher cardiovascular risk observed in stone formers. Renal stone formers are often overweight or obese. In three large prospective cohorts of nearly 250,000 individuals in the US, the RR for incident stone formation in subjects >100 kg was 1.44 in men, 1.89 in older women, and 1.92 in younger women ^17^. In subjects with metabolic syndrome (impaired fasting glucose, elevated blood pressure, dyslipidemia, and central obesity), the prevalence of nephrolithiasis is high with an increased risk of stone formation in men (OR 2.1) and in women (OR 4.9) ^18^. Idiopathic uric acid nephrolithiasis has been regarded as a renal manifestation of the metabolic syndrome because of the impaired renal production and transport of ammonia that could be related to insulin resistance ^19, 20^. Also, the risk of calcium stone formation increases with the number of features of the metabolic syndrome, although further studies are necessary to establish a clear relationship between calcium nephrolithiasis and metabolic syndrome/cardiovascular risk and to disclose the potential mechanisms ^21, 22^.

## Pathogenesis

The formation of kidney stones has been explained by different pathophysiological mechanisms ^23^. Urinary calculi originate from the formation of crystals in the urine, which is a complex solution of various solutes. When the urine becomes supersaturated due to a low urine volume or excessive excretion of solutes, crystalline formation begins. The crystals may grow gradually or aggregate. Formation, growth, and aggregation of crystals may be influenced by substances present in the urine which act as promoters or inhibitors of crystallization. A lack of crystallization inhibitors (magnesium, citrate, and macromolecules) can be the origin of kidney stone formation. Crystallization starts in the renal parenchyma in ways that are not completely known.

### Free or fixed particle theory

The increasing concentration of lithogenic solutes along the different segments of the nephron involves supersaturation conditions leading to the formation, growth, and aggregation of crystals that might get trapped in the tubular lumen and begin the process of stone formation. The phenomenon could start with crystalline aggregates free in the tubular lumen (free particle theory) or coated on the wall of the tubule (fixed particle theory). The speed of growth of the crystalline aggregates, the diameter of the different segments of the nephron, and the transit time in the nephron are crucial elements in justifying one theory or the other ^24^. Plugs of crystalline material were highlighted with histopathological examinations in the tubular lumen of patients with brushite stones or stones associated with hyperparathyroidism, renal tubular acidosis, hyperoxaluria secondary to intestinal surgery (bypass for obesity, ileal resection, or ileostomy), or cystinuria.

### Randall’s plaques

An alternative mechanism to the crystalline growth within the tubules is crystalline growth starting from plaques of calcium phosphate in the interstitium within the renal papillae. The presence of small plaques of crystalline material in the papillae of the renal calyces, which has been identified as a pre-lithiasic condition (Randall’s plaques), was described in the 1930s ^25^. In more recent years, this observation has been re-evaluated and the nature of these changes has been better investigated by endoscopic observations of the renal cavities *in vivo* or by micro-computed tomography (CT) analysis of the structure of stones. The origin of the plaques is still under discussion because they may be derived from the basement membrane of the loop of Henle ^26^ or from deeper structures such as the basal membrane of the collecting tubules and vasa recta ^27^. An intervention in the pathogenesis of Randall's plaque of interstitial cells with the capacity to transdifferentiate along the bone lineage has also been suggested ^28– 30^.

## Urinary risk factors for urolithiasis

In the 1960s and 1970s, many studies identified several possible risk factors for stone formation in the composition of the urine of renal stone formers ^31, 32^. For calcium stones (oxalate and calcium phosphate), the main risk factors were identified in high concentrations of calcium and oxalate, which are the main components of these stones, and a lower excretion of magnesium and citrate, which act as inhibitors of crystallization. In calcium oxalate stone formation, increases in urinary oxalate are more relevant than increases of urinary calcium because calcium is present in 10−20-fold higher concentrations in urine and calcium oxalate crystallization occurs in a 1:1 molar ratio. This implies that isolated increases in urinary calcium will not produce more particles ^33^, whereas increases of oxalate produced by a dietary load of oxalate may produce microliths within 24 hours, even in non-stone former subjects ^34^. On the other hand, due to the excess of urinary concentrations of phosphate (from bone and protein metabolism), an increase of urinary calcium will tend to produce calcium phosphate microliths. Inadequate urinary output is another major risk factor, whereas pH values of >7 tend to increase the crystallization of calcium phosphate. For calculi of uric acid, an excessive excretion of uric acid and undue acidic urinary pH are the most important risk factors. In fact, low urinary pH increases the concentration of the insoluble undissociated uric acid.

In some cases, these changes in urinary composition are caused by well-identifiable diseases. Excessive calcium excretion is a characteristic feature of primary hyperparathyroidism, sarcoidosis, prolonged immobilization, and other bone diseases. Hyperoxaluria can be observed in some congenital abnormalities of metabolism (primary hyperoxaluria) and some acquired forms (inflammatory bowel disease and results of bariatric surgery). Finally, some drugs cause high concentrations of calcium (loop diuretics), oxalate (orlistat), or urate (losartan); others cause increased concentrations of urinary calcium in association with increased urinary pH and lower urinary citrate (acetazolamide, topiramate, and zonisamide) or reduce the concentration of inhibitors such as citrate (thiazide diuretics and angiotensin-converting enzyme [ACE] inhibitors).

The pathogenic mechanisms for unduly urinary pH in uric acid stone formers are increased net acid excretion (NAE) and reduced renal ammonium (NH ^4+^). The production and transport of ammonia could be impaired by insulin resistance, whereas the underlying mechanism of increased acid production has still to be fully elucidated.

Infection stones are caused by definite urinary abnormalities secondary to infection by urease-producing bacteria. *Proteus* species and to a lesser extent *Klebsiella* and *Enterobacter* species present with an enzymatic activity which cleaves the urea present in the urine into ammonium and bicarbonate. The alkalinity and the high urinary concentrations of ammonium cause the crystallization of magnesium ammonium phosphate (struvite) with formation of large stones that may fill the renal cavities (staghorn stones). The infection by urease producers is often favored by congenital or acquired alterations of the urinary tract, causing stasis of the urine and leading to the appearance and maintenance of infection.

Other types of stones (cystine, xanthine, and dihydroxyadenine) are caused by specific congenital metabolic defects that cause excessive excretion of these poorly soluble substances that tend to precipitate. Finally, some stones are caused by the precipitation of medications themselves (indinavir and other antiretroviral drugs) under conditions of reduced urinary output. However, in the great majority of cases, the alterations in the composition of the urine are not associated with specific diseases and are defined as idiopathic nephrolithiasis. In these cases, the causes of the disease are related to exposure to environmental factors in genetically predisposed subjects.

## Genetics

Specific types of nephrolithiasis are clearly linked to some monogenic hereditary alterations, which account for nearly 2% of renal stone cases in adults and 10% in children ^35^. Cystinuria is a defect in tubular reabsorption of cystine and dibasic amino acids, which results in the frequent recurrent formation of stones composed of cystine. Inborn errors of the metabolism of oxalate (primary hyperoxalurias) result in the recurrent formation of calcium oxalate stones and crystal deposition in the renal parenchyma with associated progressive renal failure. Monogenic alterations of purine metabolism can also cause stones of uric acid or other purines (2,8-dihydroxyadenine or xanthine), crystal renal deposition, and progressive renal failure. A group of congenital tubulopathies affecting the convoluted proximal tubule (such as Dent's disease, Lowe syndrome, or hypophosphatemic rickets), the thick ascending limb of the loop of Henle (such as familial hypomagnesemia and Bartter's syndrome), or the distal part of the nephron (congenital distal tubular acidosis with or without hearing loss) are associated with calcium phosphate stone formation, nephrocalcinosis, extensive tubulointerstitial fibrosis, and a significant risk of progressing toward end-stage renal disease. Recurrent calcium stones associated with medullary sponge kidney (MSK) may be associated with an autosomal dominant mutation of a still unknown gene ^36^. One of the most interesting candidate genes is *GDNF*, a gene involved in renal morphogenesis ^37^. For all these diseases, it is now possible to make a precise genetic diagnosis with the identification of specific mutations; however, they are sometimes misdiagnosed or diagnosed late because the clinical presentation is not recognized ^38^. Also, in the most common idiopathic calcium stone disease, a genetic basis is very likely because only a proportion of those exposed to the same environmental risk factors present with the disease.

The familial association of idiopathic calcium stone disease has been demonstrated by numerous studies, although the specific genetic and epigenetic factors have remained less clear. Calcium stone disease seems to be a genetically heterogeneous disease related to multiple genetic factors that regulate the excretion of the different urinary risk factors. Family-based or case-control studies of single-candidate genes showed gene polymorphisms related to stone formation for calcium-sensing receptor, vitamin D receptor, Na ^+^/dicarboxylate cotransporter-1, and osteopontin ^39, 40^. A recent genome-wide association study in a large cohort of hypercalciuric stone formers from Iceland and the Netherlands identified the claudin 14 ( *CLDN14*) gene as a possible major gene of nephrolithiasis ^41^.

## Environmental factors

Dietary intake is certainly the most important environmental risk factor. Several studies of large cohorts of prospectively studied subjects showed some possible associations between the levels of intake of some nutrients and the risk of forming kidney stones. In particular, an insufficient intake of dietary calcium (<600 mg/day) can increase the intestinal absorption of oxalate with increased saturation values for urinary calcium oxalate and the risk of calcium oxalate stone formation ^42^. Other possible risk factors that have been identified include excessive intake of salt and proteins ^43^. The potential role of the acid load of the diet, related to the content in animal protein and the relationship between intake of calcium, magnesium, and potassium and that of chlorine and phosphates, seems to play an important role in causing a state of subclinical chronic acidosis and the consequent excessive mobilization of calcium from bone and its excretion in the urine ^44– 47^. For this reason, the intake of vegetables in association with the reduction in salt is encouraged in stone-forming patients. Consumption of sugar-sweetened soda and punch is associated with a higher risk of stone formation, whereas consumption of coffee, tea, beer, wine, and orange juice is associated with a lower risk ^48, 49^.

### Environment and lifestyle

People living in hot climates or who are exposed to high temperatures at work are at increased stone risk due to reduced urinary diuresis. Physical inactivity appears to be a risk factor for urinary stones, while moderate and constant physical activity seems sufficient to reduce the risk of forming kidney stones. However, the link between physical activity and risk of kidney stones is still uncertain and needs to be further investigated ^50^.

## Treatment of kidney stones

Over the last 40 years, the methods of stone removal from the urinary tract have benefited from technological innovations that have revolutionized the treatment of the disease and reduced its morbidity. Extracorporeal shock wave lithotripsy and endoscopic techniques of intracorporeal lithotripsy (percutaneous and retrograde) have made the treatment of kidney stones minimally invasive and easily repeatable. Conversely, medical treatment was not substantially improved. Despite increased knowledge about the pathogenesis and predisposing diseases and risk factors, the current modality of dietary and pharmacological treatment and their results are still unsatisfactory.

### Fluid intake

A high fluid intake is widely recognized as the cornerstone of prevention of all forms of stones, although only one randomized trial has confirmed the effectiveness of this preventive measure (RR = 0.45) ^51^. A reduction in the consumption of soft drinks significantly reduces the risk of lithiasis (RR = 0.83) ^52^.

### Diet

Numerous studies have shown the effects of different types of nutritional intervention on urinary risk factors for the formation of kidney stones, but the evidence from randomized trials is still low and of uncertain meaning ^53^. The effectiveness of protein restriction has been evaluated in several studies that still do not allow definitive conclusions to be made owing to the heterogeneity of experimental designs. Protein restriction alone was compared with that of a diet with high fiber content without demonstrating significant differences ^54^. Conversely, protein restriction in combination with salt restriction and a calcium intake normalized according to the levels of intake recommended for the general population was more effective than a low-calcium diet ^55^. However, in this last study, the effect of the reduction in protein intake cannot be distinguished from that of salt restriction and, in turn, the overall effect of this diet cannot be well estimated because the control group was on a diet potentially favoring the formation of stones. Other controlled studies have used multiple variables and dietary interventions that make interpretation difficult. Randomized trials evaluating more specific and targeted interventions are required to obtain more robust information on the effectiveness of dietary treatment of kidney stones. However, the unpredictable nature of nephrolithiasis, its complexity and heterogeneity, and, last but not least, lack of interest from pharmaceutical companies make these studies very difficult to perform.

### Pharmacological prevention

The treatment and prophylaxis of kidney stones find their foundations in numerous studies dating back to over 30 years ago. The mechanisms of action and effects of several pharmacological measures on several urinary risk factors have been well studied, but unfortunately clinical evidence from randomized controlled studies is still scarce ^53^. Uric acid stones can be dissolved with an oral alkalinizing therapy by means of potassium bicarbonate or potassium/sodium citrate ^56^. The prevention of this type of stone is based on the long-term taking of citrate. Calcium stone formation can be prevented by the use of different drugs with different mechanisms of action, such as thiazide diuretics, allopurinol, phosphates, and potassium citrate. A meta-analysis of randomized controlled trials ^53^ showed that thiazide diuretics (five studies) may reduce the risk of stone formation by 48% (RR = 0.52) and that the association with allopurinol (RR = 0.79) or with citrates (RR = 0.94) does not significantly increase the effectiveness of these drugs. The use of thiazides is, however, limited by the fear of side effects in the long term. The major concerns about their use arise from their tendency to cause hypokalemia, impaired glucose tolerance, and increases in serum cholesterol and serum uric acid ^57^. Citrates (four studies) may reduce the risk of recurrence by 75% (RR = 0.25) and allopurinol (two studies) of 41% (RR = 0.59) ^53^. Citrates are devoid of potentially serious side effects and may have a favorable impact on low bone density, which is frequently observed in the calcium stone patient, but are poorly tolerated for their digestive effects. On the other hand, although in rare cases, allopurinol may cause severe hypersensitive reactions ^58^. Finally, the results of the use of magnesium (one study) were not significant. In conclusion, although there are several options for the pharmacological prevention of nephrolithiasis, there is no ideal drug that is both risk free and well tolerated. Finally, compliance to a prolonged pharmacological treatment remains a serious limitation of all forms of long-term treatment for a chronic disease, for which treatment effectiveness is conditioned by an efficient follow-up organization.

## Conclusions

The most important future objectives of renal stone research are epidemiological studies that investigate simultaneously the genetic aspects and the diets of stone patients, studies aimed at correlating the morphological endoscopic and histological aspects of renal papillae with stone composition and chemical composition of urine from the same stone-forming patients, and large-scale randomized studies that evaluate the long-term effects of dietary modifications and pharmacological treatments for the prevention of recurrent stones.
